# Intentions for post-abortion contraceptive use among women who received abortion services in health facilities of Harar city and Dire Dawa city

**DOI:** 10.3389/fgwh.2025.1507022

**Published:** 2025-03-20

**Authors:** Meron Degefa, Nega Assefa, Merga Deresa, Dawit Abebe, Sinetibeb Mesfin

**Affiliations:** ^1^ School of Nursing and Midwifery, College of Health and Medical Sciences, Haramaya University, Harar, Ethiopia; ^2^ School of Nursing and Midwifery, College of Health and Medical Sciences, Jigjiga University, Jigjiga, Ethiopia

**Keywords:** intention, post-abortion care, contraceptive, eastern Ethiopia, family planning

## Abstract

**Background:**

A woman's specific beliefs about contraceptives influence her engagement and adherence to these methods. The intention to use post-abortion contraceptive methods is a critical aspect of reproductive health, particularly for women who have undergone abortion procedures. The use of less effective contraceptive methods, inconsistent usage, and discontinuation significantly contribute to unintended pregnancies, which are a primary cause of abortion. Studies indicate low contraceptive utilization and a high prevalence of abortion in the study area. Therefore, this study aimed to assess the intention to use post-abortion contraceptives and the associated factors among women visiting health facilities for abortion services, utilizing a behavioral model.

**Objective:**

To assess intention to use post abortion contraceptive use and associated factors among women visiting health facilities for abortion services in Harari region and Dire Dawa City Administration, eastern Ethiopia.

**Methods:**

An institutional-based cross-sectional study was conducted among 609 women who received abortion services in the Harari region and Dire Dawa city administration health facilities. All women who sought abortion services at these health facilities during the study period were included in the research. Data were collected using a structured, interviewer-administered questionnaire. Binary logistic regression was employed to predict the association between dependent and independent variables. Variables with a *p*-value of less than 0.05 were considered statistically significant.

**Result:**

The overall prevalence of intention to use post abortion contraceptive of women came for abortion service was 74.7% (95% CI: 71.3–78.2). Women who had contraceptive use history [AOR 2.580; 95%CI (1.216–5.473)], no pregnancy plan within the next two years [AOR 2.859; 95%CI (1.451–5.635)], positive attitude [AOR 3.335; 95%CI (1.831–6.077)], high level of subjective (perceived) norm [AOR 3.348; 95%CI (1.805–6.210)], high level of perceived behavioral control [AOR 6.784; 95%CI [(3.650–12.607)] were positively associated with intention to use post abortion contraceptive. Women who were divorced [AOR 0.149; 95%CI (0.039–0.578)] and had wanted pregnancy abortion [AOR 0.336; 95%CI (0.153–0.735)] variables were negatively associated with intention to use post abortion contraceptive.

**Conclusion:**

This study revealed that more than two-thirds of the participants expressed an intention to use contraception following an abortion. Healthcare providers should offer comprehensive education and counseling on contraceptive options for women post-abortion. Furthermore, it is essential to provide personalized counseling to address each woman's unique needs and concerns regarding contraceptive choices.

## Introduction

Every year, approximately 121 million women worldwide experience unintended pregnancies, and around 73.3 million of them undergo abortions (1). The use of less effective contraceptive methods, inconsistent or infrequent usage, and discontinuation of contraceptives significantly contribute to unintended pregnancies, which are a primary cause of abortion (2). The World Health Organization (WHO) guidelines recommend that a woman should wait at least six months after an abortion before attempting to conceive again (3). Shorter intervals of less than six months between a miscarriage or induced abortion and a subsequent pregnancy are associated with an increased risk of adverse maternal and perinatal outcomes (4, 5).

The intention to use post-abortion contraceptive methods is a critical aspect of reproductive health, particularly for women who have undergone abortion procedures (6). A woman's specific beliefs about contraceptives influence her engagement and adherence to these methods (7). The Theory of Planned Behavior (TPB) is an effective predictor of intention and behavior. It helps us understand why people make certain decisions, particularly in health-related contexts. According to TPB, our intentions are shaped by how we feel, what others think, and how much control we perceive over the situation (8). The theory comprises attitude, subjective norm, and perceived behavioral control. It suggests that that if a person has a strong intention to engage in a behavior, they are more likely to perform it (9).

The Ethiopian Ministry of Health has made significant progress in expanding comprehensive abortion care. This effort includes the development and dissemination of national guidelines for the provision of legal and safe abortion services, established in 2006 in accordance with World Health Organization standards (10). Furthermore, the ministry has supported the expansion of services provided by the private sector and has integrated safe abortion and post-abortion contraception into existing reproductive health services.

Despite these efforts, the annual abortion rate in Ethiopia increased from 22 per 1,000 women in 2008 to 24 per 1,000 women in 2019 among those aged 15–49. The rate is notably higher in certain areas, with Addis Ababa reporting 49 per 1,000 and the towns of Harar and Dire Dawa reporting 184 per 1,000 women, significantly surpassing other regions (8). A systematic review by Kumsa H et al. found that the pooled prevalence of pregnancy termination in Ethiopia was 21.52% (95% CI: 15.01%–28.03%).

As far as the investigator is aware, some studies in Ethiopia have been conducted from the perspective of intention regarding contraceptive use (11, 12). However, these studies did not incorporate the Theory of Planned Behavior, thus leaving out important factors that are necessary for measuring intention-driven behavior, which plays a vital role in understanding planned actions. In addition, research indicates that there is low contraceptive utilization and a high prevalence of abortion in the study area. Therefore, this study aims to identify various factors that encourage women to consider contraceptive methods, as well as those that may hinder their intentions.

## Methodology

### Study design, setting and period

A facility-based cross-sectional study was conducted from March 1 to April 6, 2022, in the Harar region and the Dire Dawa city administration health facilities. Harar is one of the cities in eastern Ethiopia, situated approximately 500 kilometers from the national capital, Addis Ababa, at an elevation of 1,885 meters. According to 2013 population projection Harari region has an estimated total population of 226,000 consisting of 114,000 male and 112,000 females. 44.7% of the population is estimated to be rural inhabitants, while 55.3% are urban dwellers.The city is has two public hospitals, eight health centers, and 27 health posts. Additionally, there are two private hospitals and a non-profit family guidance health institution.

Dire Dawa city administration is situated approximately 515 kilometers from the national capital, Addis Ababa, at an elevation of 1,275 meters. According to the 2013 population projection, the Dire Dawa city administration had an estimated total population of 427,000, with 214,000 males and 213,000 females. Of the total population, approximately 37.1% were rural residents, while 62.9% lived in urban areas. The city is home to two public hospitals, 15 health centers, and 34 health posts. Also, there are eight non-governmental health institutions.

### Sample size determination and sampling technique

The sample size for both objectives was determined using single and double population proportion formulas, with the larger sample size selected for this study. For the first objective, the sample size was calculated using the single population proportion formula, which considered a 73.5% prevalence of the intention to use post-abortion contraceptives based on a study conducted in Gondor, Ethiopia (12). A 95% confidence level and a 5% margin of error (d) were applied, resulting in a calculated sample size of 299.

The sample size for the second specific objective was determined by considering factors significantly associated with the outcome variable, a two-sided confidence level of 95%, a margin of error of 5%, a power of 80%, and a ratio of exposed to unexposed of 1:1, using STATCALC from Epi Info Version 7. Based on these assumptions, the final sample size was 618 after accounting for a 10% non-response rate.

The study purposefully included a range of health facilities in both Harar and Dire Dawa based on patient flow. These facilities consisted of governmental hospitals, NGO health centers, and public health institutions to ensure a comprehensive representation of abortion care services. Reports from the regional health bureaus of Dire Dawa and Harar were used to estimate the number of women seeking abortion care over one month and one week, with a total of 623 women projected to visit the selected facilities during that time.

To determine these estimates, a term report was obtained from each regional health bureau and divided by three to calculate the average number of women accessing abortion care in one month. This monthly estimate was further divided by four to approximate the weekly patient flow. The estimated numbers varied across facilities, but collectively 623 women were expected to receive abortion services over the study period. All women visiting the selected health facilities for abortion care were interviewed until the target sample size was achieved.

### Study population and eligibility criteria

All women who came to selected health facilities for abortion service during the study period constituted the study population. Women who visited these facilities for abortion service during the study period were included in the study, while those in critical condition were excluded.

### Data collection tools and procedure

Data were collected using a structured, pre-tested, interviewer-administered questionnaire developed through a review of various literature. After receiving three days of training on the study's purpose, ethical considerations, and data collection techniques, ten BSc midwives conducted face-to-face interviews to collect the data.

### Operational definitions

Abortion- defined as termination of a pregnancy before 28 weeks of gestation (13).

Positive Attitudes—when participants summed scored value equal or greater than the median value of summed score 5 attitude related questions (14).

Induced abortion—defined as an abortion that occurs with any medical or surgical intervention (13).

Intended to use contraceptive- those participants who answered yes for post abortion contraceptive intention item (14).

High level of Perceived behavioral control—When participants’ summed score values were equal to or greater than the median value of the summed scores from six perceived behavioral control-related questions (14).

High level of Subjective norm—When participants’ summed scores were equal to or greater than the median value of the summed scores from 11 subjective norm-related questions (14).

Spontaneous abortion—defined as an abortion occurring without any medical or surgical means to empty uterus (13).

Unintended pregnancy is a pregnancy that is either mistimed (occurred earlier than desired) or unwanted (occurred when no children or no more children were desired) at the time of conception (15).

### Data processing and analysis

The data were coded, entered, and cleaned using Epi-Data version 3.1 software, and were subsequently exported to SPSS version 20 for analysis. First, descriptive analysis was conducted for each subgroup, followed by the calculation of summed scores for three constructs: attitude, perceived norm (subjective norm), and perceived behavioral control. The median value for each summed score was determined for the three components. The attitude variable was categorized into positive and negative attitudes. A woman who scored a median value of 18 or above was classified as having a positive attitude. The subjective (perceived) norm and perceived behavioral control variables were categorized as low level and high level. Within the subjective (perceived) norm subdivision, there were eleven questions; thus, a woman who scored a median value of 38 or above was classified as having a high level of subjective (perceived) norm. Women who scored a median value of 22 or above out of six perceived behavioral control-related items were classified as having a high level of perceived behavioral control. Finally, for the outcome variable (intention), women who answered “yes” to the intention-related question were assigned a code of “1” (indicating they intended to use it), while those who answered “no” were assigned a code of “0” (indicating they did not intend to use it). Univariate analysis, including simple frequencies, measures of central tendency, and measures of variability, was employed to describe the characteristics of the participants.

Bivariate analysis, including crude odds ratios with 95% confidence intervals, was employed to examine the association between each independent variable and the outcome variable using binary logistic regression. Multi-collinearity was assessed using the Variance Inflation Factor (VIF) and tolerance tests. All variables that yielded a *p*-value of less than 0.25 in the bivariate analysis were considered candidates for multivariable logistic regression analysis. Associations with *p* < 0.05 in the multivariable logistic regression were declared as statistically significant.

### Ethical consideration

Ethical clearance was obtained from the Haramaya University College of Health and Medical Sciences Institutional Health Research Ethical Review Committee (IHRERC). The study's purpose, procedures, duration, potential risks, and benefits were clearly explained to the participants in the local language. Subsequently, individual informed, voluntary, written, and signed consent was obtained. We assured respondents that their information would remain confidential by not asking for their names during data collection. They were also informed that they could choose not to participate or stop at any time if they felt uncomfortable.

## Result

### Socio-demographic characteristics

In this study, a total of 609 women participated, resulting in a response rate of 98.5% from the sampled 618. Nine participants were excluded due to incomplete data. The mean age of the women was 25.09 years (±5.17 SD). One-third of the women, totaling 203 (33.3%), fell within the age group of 20–24 years. Half of the women, 321 (52.7%), were married. Among the married women, 50.5% had been married for five years or less. Nearly half of the participants, 277 (45.5%), identified as Muslim. Regarding educational attainment, 216 (35.5%) had completed preparatory education or higher. Two hundred sixty-three women (43.2%) belonged to the Oromo ethnicity, and the majority, 479 (78.7%), resided in urban areas. Approximately one-third of the participants were housewives (Table 1).

**Table 1:** Socio-demographic characteristics of women seeking abortion services in health facilities of the Harari region and Dire Dawa city administration, eastern Ethiopia.

Variables	Categories	Number	Percentage (%)
Age(*n* = 609)	15–19	92	15.1
	20–24	203	33.3
	25–29	183	30.0
	30–34	94	15.4
	≥35	37	6.1
Marital status (*n* = 609)	Single	253	41.5
	Married	321	52.7
	Divorced	35	5.7
Duration of marriage (*n* = 321)	<5year	162	50.5
	6–10 years	100	31.2
	>10 years	59	18.4
Religion (*n* = 609)	Muslim	277	45.5
	Orthodox	222	36.5
	Other*	110	18
Level of education (*n* = 609)	No formal education	89	14.6
	Primary(1–8)	123	20.2
	Secondary(9–10)	181	29.7
	Preparatory & above	216	35.5
Ethnic group (*n* = 609)	Oromo	263	43.2
	Amhara	187	30.7
	Somali	46	7.6
	Harari	34	5.6
	Tigray	38	6.2
	Other**	41	6.7
Residence (*n* = 609)	Rural	130	21.3
	Urban	479	78.7
Occupation (*n* = 609)	Housewife	194	31.9
	Gov'tal employer	91	14.9
	Some job/private	117	19.2
	Student	184	30.2
	Other***	23	3.8

Other*: Protestant, Catholic and Wakefeta, Other**: Gurage and Hadiya Other***: Farmer, Unemployed, Home made and Prostitute.

### Past obstetrics and contraceptive history

Half of the participants, 309 (50.7%), had no children. The majority of the women, 506 (83.1%), reported no previous history of abortion. Most of the women, 526 (86.4%), had received some form of general information about contraceptives before the study. Among these, approximately 400 (65.7%) women had more detailed knowledge about specific contraceptive methods, with 397 (75.5%) aware of pills and 392 (74.5%) familiar with injectable methods. Regarding contraceptive use history, more than half of the respondents, 398 (65.4%), had previously used contraceptives, with injectable being the most commonly used method at 181 (45.5%). About one-third of the women had no history of contraceptive use, and the most common reason for not using contraceptives was the desire to have children, reported by 39 (18.6%) of the participants (Table 2).

**Table 2:** Past obstetric and contraceptive history of women seeking abortion services in health facilities of the Harari region and Dire Dawa city administration, eastern Ethiopia.

Variable	Categories	Number	Percentage (%)
Previous obstetrics related variables			
Number of children (*n* = 609)	No child	309	50.7
	1–2	163	26.8
	≥3	137	22.5
Previous abortion history (*n* = 609)	Yes	103	16.9
	No	506	83.1
Previous contraceptive history related factors			
Information about contraceptive (*n* = 609)	Yes	526	86.4
	No	83	13.6
Source of information (*n* = 526)	TV	246	46.8
	Public health institution	317	60.3
	Private health institution	61	11.6
	Peer	213	40.5
	Other*	18	3.4
Type of contraceptive that have information about (*n* = 526)	Oral contraceptive	397	75.5
	Injectable	392	74.5
	Implanon	308	58.6
	Traditional	22	4.2
	IUCD	81	15.4
	Condom & other barrier meth	21	4
History of contraceptive use (*n* = 609)	Yes	398	65.4
	No	211	34.6
Type of Contraceptive used (*n* = 398)	Pills	160	40.1
	Injectable	181	45.5
	Implanon	92	23.1
	Other**	21	5.2
Reasons for not using contraceptives (*n* = 211)	Insufficient knowledge	33	15.6
	Fear of side effects	13	6.2
	Cultural or religious	29	13.7
	Being student	11	5.2
	Being not married	33	15.6
	Desire to have a child (recently married)	39	18.5
	Start sex near time	25	11.8
	Not anticipating pregnancy	12	5.7
	Other***	16	7.6

Other*: Leaflet, School and Family, Other**: Traditional, IUCD and Barrier Methods Other***: Husband Refusal, Do not want it and Got raped.

### Present obstetrics and service care history

More than half of the respondents, 367 (60.3%), were from public health facillities. Regarding their pregnancy desires, 357 (58.6%) reported that their pregnancies were unplanned. Three hundred ninety-three (64.5%) of uterine evacuations were performed through medical termination. Approximately half, 262 (43.0%), of pregnancy terminations were conducted by midwifery professionals. Out of the respondents, 413 (67.8%) indicated that they have no pregnancy plans within the next two years (Table 3).

**Table 3:** Current obstetric and service care history of women seeking abortion services: characteristics of women who came for abortion services in health facilities of the Harari region and Dire Dawa city administration, eastern Ethiopia.

Variable	Categories	Number	Percentage (%)
Type of institution (*n* = 609)	Public	367	60.3
	NGO	242	39.7
Types of uterine evacuation performed (*n* = 609)	Medical Abortion	393	64.5
	MVA/EMA(surgical)	195	32.0
	Both	21	3.4
Profession of service providers (*n* = 609)	Doctor	113	18.6
	Health officer	53	8.7
	Midwifery	262	43.0
	Nurse	181	29.7
Planning for pregnancy within the next two years (*n* = 609)	Yes	196	32.2
	No	413	67.8

### Attitude, subjective (perceived) norm and perceived behavioral control towards contraceptive

**Figure 1:**
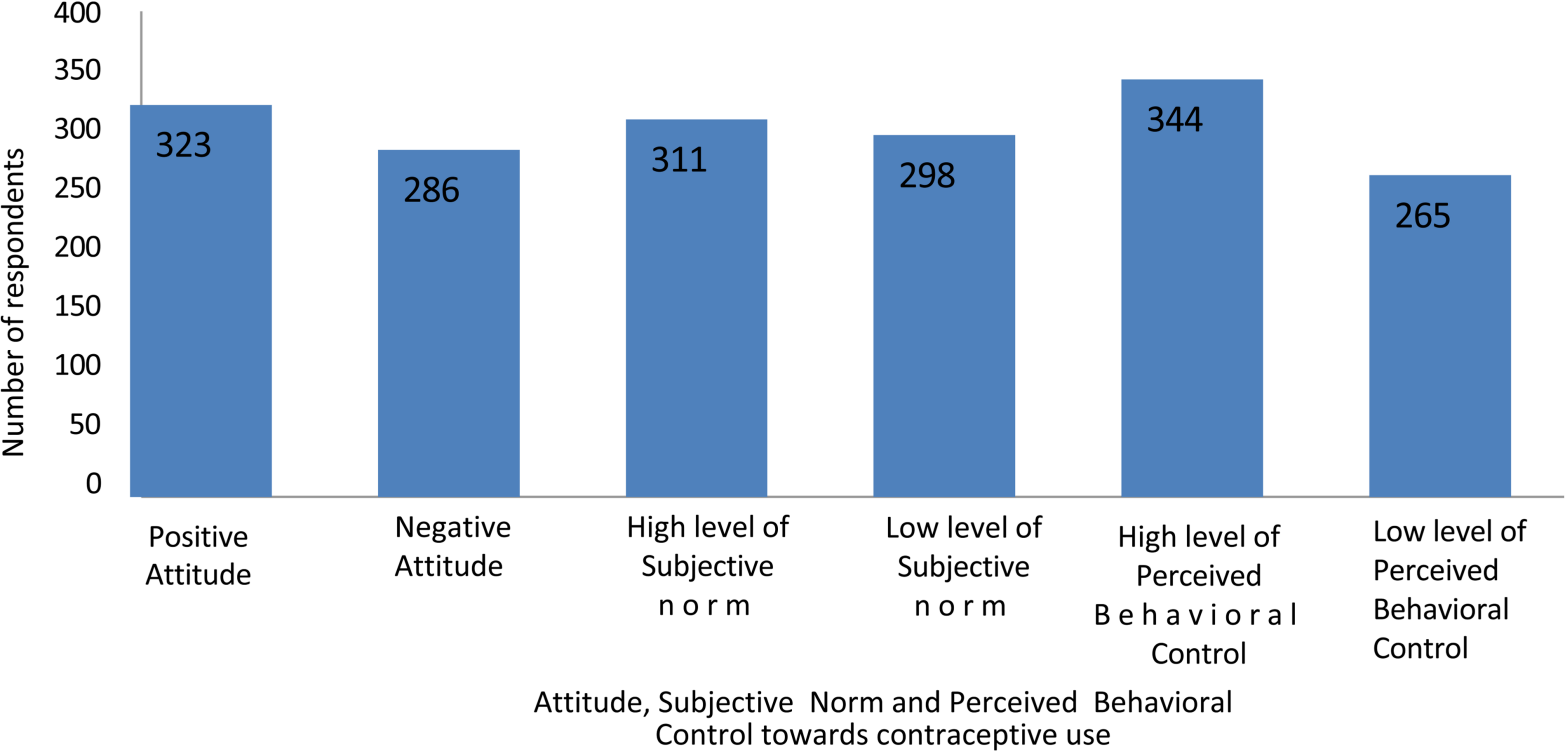
Attitude, level of subjective (perceived) norm and level of perceived behavioural control of women came for abortion service (*n* = 609) in health facilities of Harari region and Dire Dawa city administration, eastern Ethiopia.

Attitude, subjective norm, and perceived behavioral control were measured using the components of the Theory of Planned Behavior. The median scores for each summed score were calculated, resulting in median scores of 18 for attitude, 38 for subjective (perceived) norm, and 22 for perceived behavioral control, respectively. More than half of the women, 323 (53%), scored at or above the median, indicating a positive attitude toward contraception. Among these respondents, 311 (51.1%) exhibited a high level of subjective (perceived) norm, while 287 (47.1%) demonstrated a high level of perceived behavioral control (Tables 4–6; Figure 1).

**Table 4:** Number of responses to five attitude-related items from women seeking abortion services in health facilities of the Harari region and Dire Dawa city administration, eastern Ethiopia.

Attitude related items	Strongly disagree	Disagree	Neutral	Agree	Strongly agree
I don't believe that birth control will have negative side effects.	36	186	131	218	38
I believe that birth control will be more beneficial than harmful for me.	14	78	89	342	86
I believe I might get pregnant if I don't use birth control correctly.	17	27	94	346	125
I would feel upset if I became pregnant in the next year.	74	91	58	192	194
It is important is to use birth control when there is no often sex.	55	144	166	189	55

**Table 5:** Number of responses of eleven subjective (perceived) norm related items of women came for abortion service in health facilities of Harari region and Dire Dawa city administration, eastern Ethiopia.

Items of subjective (perceived) norm	Possible answers				
	Strongly disagree	Disagree	Neutral	Agree	Strongly agree
My husband or boyfriend support me in using birth control	40	124	105	287	53
Health professionals support me in using birth control	14	21	91	378	105
My family support me in using birth Control	66	178	139	189	37
My friends support me in using birth Control	32	112	172	247	46
I do not want to do as all what my boyfriend husband thinks I should do	29	160	92	295	33
I do not want to do as all what Health professionals thinks I should do	19	150	105	295	40
I do not want to do as all what my family thinks I should do	30	195	108	249	27
I do not want to do as all what my friends thinks I should do	29	141	145	258	36
Health professionals use Contraceptives	11	19	199	280	100
My friends use contraceptives	19	67	150	303	70
My family use contraceptives	57	94	129	286	43

**Table 6:** Number of responses of six perceived behavioral control related items of women came for abortion service (*n* = 609) in health facilities of Harari region and Dire Dawa city administration, eastern Ethiopia.

Items of perceived behavioral control	Strongly disagree	Disagree	Neutral	Agree	Strongly agree
I'm certain I'll use birth control for the next year.	73	77	67	283	109
I'm confident that I'll be proactive about using birth control.	50	88	99	267	105
I'll find it easy to use birth control correctly.	24	90	117	297	81
I'm sure I could stand firm with my husband or boyfriend if he doesn't want me to use contraception.	34	103	135	264	73
It's really important for me to prevent pregnancy at this time.	71	89	53	237	159
I want to accomplish things like finishing school, getting a job, and earning more money before having a child or my next child.	75	86	70	219	159

### Intention towards contraceptive

More than two-thirds of the participants, 455 (74.7%), reported an intention to use post-abortion contraception (95% CI: 71.3–78.2). Among those with this intention, 255 women (41.9%) planned to use Implanon. Conversely, 154 women (25.3%) expressed no intention to use contraceptives after their abortion; of these, 89 women (57.8%) indicated that they did not intend to engage in family planning because they desired to have children.

### Factors affecting intention to use post abortion contraceptive

In the bivariable model, intention to use post abortion contraceptive was significantly associated with marital status, duration of marriage, level of education, residence, occupation, number of child, information about contraceptive, contraceptive use history, type of healthcare institutions, desire for pregnancy, Clinical service provider profession, plan for pregnancy within the next two years, attitude towards contraceptive, subjective (perceived) norm and perceived behavioral control.

Out of those associated variables in bivariable analysis; marital status, contraceptive use history, desire for pregnancy, plan for pregnancy within the next two years, attitude, subjective (perceived) norm and perceived behavioural control were maintain their significant association with intention towards post abortion contraceptive use in multivariable analysis.

Divorced women had an 85% lower likelihood of intending to use post-abortion contraception compared to single women [Adjusted Odds Ratio (AOR) 0.149; 95% Confidence Interval (CI) (0.039–0.578)]. Women with a history of contraceptive use were 2.6 times more likely to intend to use post-abortion contraception than those without such a history [AOR 2.580; 95% CI (1.216–5.473)]. Women who had abortions for unplanned pregnancies were 66% less likely to intend to use post-abortion contraception [AOR 0.336; 95% CI (0.153–0.735)] compared to those who had abortions for unwanted pregnancies. Women who did not plan for pregnancy within the next two years had increased odds of intending to use post-abortion contraception [AOR 2.859; 95% CI (1.451–5.635)] compared to those who had a plan. Women with a positive attitude towards contraception were three times more likely to intend to use post-abortion contraception [AOR 3.335; 95% CI (1.831–6.077)], while those with a high level of subjective (perceived) norms were also three times more likely to intend to use it [AOR 3.348; 95% CI (1.805–6.210)]. Additionally, women with a high level of perceived behavioral control were six times more likely to intend to use post-abortion contraception [AOR 6.784; 95% CI (3.650–12.607)] compared to those with negative attitudes, low levels of subjective norms, and low levels of perceived behavioral control, respectively (Table 7).

**Table 7:** Factors associated with the intention to Use post-abortion contraceptives Among women seeking abortion services in health facilities of the Harari region and Dire Dawa city administration, eastern Ethiopia.

Variables		Intended (%)	Not intended (%)	COR	95% CI for COR	AOR	95% CI for AOR
Marital status	Single	228 (90.1)	25 (9.9)	1.00		1	
	Married	200 (62.3)	121 (37.7)	0.181	(0.113–0.290)	0.318	(0.100–1.017)
	Divorced	27 (77.1)	8 (22.9)	0.370	(0.152–0.902)	**0.149**	(0.039–0.578)*
Level of education	No formal	49 (55.1)	40 (44.1)	1.00		1	
	Primary (1–8)	77 (62.6)	46 (37.4)	1.366	(0.785–2.380)	0.959	(0.400–2.301)
	Secondary(9–10)	152 (84)	29 (16)	4.279	(2.404–7.614)	0.915	(0.340–2.464)
	Collage	177 (81.9)	39 (18.1)	3.705	(2.153–6.375)	0.688	(0.232–2.047)
Residence	Rural	69 (53.1)	61 (46.9)	1.00		1	
	Urban	386 (80.6)	93 (19.4)	3.669	(2.430–5.541)	1.255	(0.596–2.641)
Occupation	Housewife	116 (59.8)	78 (40.2)	1.00		1	
	Governmental	68 (74.7)	23 (25.3)	1.988	(1.143–3.456)	1.456	(0.538–3.937)
	Private	91 (77.8)	26 (22.2)	2.353	(1.397–3.966)	0.642	(0.278–1.485)
	Student	162 (88)	22 (12)	4.951	(2.915–8.410)	0.696	(0.213–2.268)
	Other	18 (78.3)	5 (21.7)	2.421	(0.863–6.791)	0.437	(0.069–2.753)
Number of children	No child	246 (79.6)	63 (20.4)	1.00		1	
	1–2	113 (69.3)	50 (70.7)	0.579	(0.375–0.892)	1.729	(0.698–4.316)
	≥3	96 (70.1)	41 (29.9)	0.6	(0.379–0.949)	1.971	(0.742–5.236)
Information about contraception	Yes	416 (79.1)	110 (20.9)	4.267	(2.641–6.892)	0.890	(0.365–2.170)
	No	39 (47.0)	44 (53.0)	1.00		1	
Contraceptives use history	Yes	335 (84.2)	63 (15.8)	4.032	(2.749–5.914)	**2.580**	(1.216–5.473)*
	No	120 (56.9)	91 (43.1)	1.00		1	
Type of institution	Public	249 (67.8)	118 (32.2)	1.00		1	
	NGO	206 (85.1)	36 (14.9)	2.712	(1.788–4.112)	1.3,334	(0.587–3.029)
Desire for pregnancy	Unwanted	318 (89.1)	39 (10.1)	1.00		1	
	Wanted	93 (47)	105 (53)	0.109	(0.70–0.168)	**0.336**	(0.153–0.735)*
	Mistimed	44 (81.5)	10 (18.5)	0.540	(0.252–1.157)	0.728	(0.264–2.006)
Profession	Doctor	57 (50.4)	56 (49.6)	1.00		1	
	Health officer	46 (86.8)	7 (13.2)	6.456	(2.687–15.51)	0.575	(0.163–2.026)
	Midwife	208 (79.4)	54 (20.6)	3.784	(2.353–6.085)	1.163	(0.575–2.355)
	Nurse	144 (79.6)	37 (20.4)	3.824	(2.282–6.407)	0.882	(0.328–2.372)
Plan for pregnancy For the next 2 years	Yes	89 (45.4)	107 (54.6)	1.00		1	
	No	366 (88.4)	47 (11.4)	9.362	(6.189–14.16)	**2.859**	(1.451–5.635)*
Attitude	Positive	294 (91)	29 (9)	7.871	(5.032–12.31)	**3.335**	(1.831–6.077)**
	Negative	161 (56.3)	125 (43.7)	1.00		1	
Subjective norm	High level	280 (90)	31 (10)	6.348	(4.102–9.826)	**3.348**	(1.805–6.210)**
	Low level	175 (58.7)	123 (41.3)	1.00		1	
Perceived behavioral control	High level	324 (94.2)	20 (5.8)	16.57	(9.933–27.65)	**6.784**	(3.650–12.607)**
	Low level	131(49.4)	134(50.8)	1.00		1	

Other: Nomads, Prostitute.

* Significant with *P* < 0.05 and **Significant with *P* < 0.001.

## Discussion

This study examined the association between various factors affecting women's intention to use post-abortion contraceptives in Eastern Ethiopia. The overall intention to use post-abortion contraceptives was found to be 74.7% (95% CI: 71.3–78.2). This finding aligns with results from studies conducted in Gambela (74.4%), and Gondar (73.5%) in Ethiopia (11, 12). However, it is slightly higher than the findings from studies in China (42.5%) (14, 16). The differences may be attributed to variations in the socio-demographic characteristics of the respondents; a majority of participants in this research had a history of contraceptive use, and this study focused on general contraceptive intention, whereas the study conducted in China specifically examined intentions toward long-acting contraceptives. Conversely, the intention rate was lower than that reported in studies from Nepal (83%), India (100%), Tanzania (89%), and Addis Ababa (90.6%) (17–19). These discrepancies may be due to variations in sample sizes and the predominance of respondents from urban regions in the in the aforementioned studies. Urban populations might have better access to healthcare services, education, and information about family planning, which could influence their intention rates.

Divorced women exhibited an 85% lower likelihood of intending to use contraceptives after an abortion compared to single women [AOR 0.149; 95% CI (0.039–0.578)]. This finding aligns with studies conducted in Gambela and Gondar, Ethiopia (11, 12). This may be attributed to the belief among many divorced women that they will not engage in sexual intercourse again, leading to a decreased concern about unwanted pregnancies (20).

Similarly to the study conducted in Nepal (14), women with a history of contraceptive use were more likely to intend to use contraceptives post-abortion compared to those without such a history [AOR 2.580; 95%CI (1.216–5.473)]. This may be attributed to the fact that individuals with prior experience are more familiar with contraceptive methods, which can increase their perceived need and likelihood of future use (21).

Women whose pregnancies were wanted but ended in abortion were less likely to use post-abortion contraceptives [AOR 0.336; 95% CI (0.153–0.735)] compared to women whose pregnancies were unwanted. Similarly, studies conducted in Gondar and Addis Ababa, Ethiopia, found that women with unwanted pregnancies were more likely to adopt contraceptives after abortion than those with wanted pregnancies (11, 13). This may be because women who desire a pregnancy typically plan to have a child. Therefore, they may seek to regain their fertility as quickly as possible in order to conceive again.

Women who did not have a pregnancy plan for the next two years were 2.9 times more likely to intend to use post-abortion contraceptives [AOR 2.859; 95% CI (1.451–5.635)]. This study aligns with research conducted in Adigrat, Ethiopia, and China (16, 22). This may be attributed to their increased awareness of the potential risks associated with unplanned pregnancies (23).

The Theory of Planned Behavior (TPB) defines attitude as the degree to which someone feels positively or negatively about doing a specific behavior. In this study, women who held a positive attitude toward contraception were 3.3 times more likely to intend to use post-abortion contraceptives compared to those who exhibited a negative attitude [AOR 3.335;95% CI (1.831–6.077)]. Similar findings have been reported in research from Nepal (14). This may be because if someone has a positive attitude towards a particular behavior, they are more likely to possess strong internal motivation to engage in that behavior. Positive attitudes often lead to stronger intentions to perform a behavior, as outlined by the TPB.

Inline to the findings of a study conducted in Nepal (14), the present study demonstrated that women with high levels of subjective (perceived) norms were 3.348 times more likely to intend to use post-abortion contraceptives than those with low levels of subjective norms. This indicates that when women feel supported by their social circles or experience less fear of judgment, they are more likely to consider using contraceptives. These results align with the Theory of Planned Behavior, which emphasizes the influence of social expectations on shaping intentions and behaviors (24, 25).

This study found that women with higher perceived behavioral control had stronger intentions to use post-abortion contraceptives compared to their counterparts with lower levels of control. A strong sense of perceived behavioral control indicates that women feel more capable of managing the behavior, which in turn boosts their motivation to take action regarding contraception. This aligns with the Theory of Planned Behavior, which highlights the crucial role of feeling capable in influencing intentions (24, 25).

## Conclusion

The findings indicate that there is relatively good magnitude of intention towards contraceptive after abortion as compared to other study. Marital status, contraceptive use history, desire for pregnancy, plan for pregnancy within the next two years, attitude towards contraceptive, level of subjective norm and level of perceived behavioral control were independent factors of women's intention towards post abortion contraceptive at Harari region and Dire Dawa city administration health institutions. Healthcare providers should offer comprehensive education and counseling on contraceptive options for women following an abortion. Furthermore, it is crucial to provide personalized counseling to address each woman's unique needs and concerns regarding contraceptive choices.

## Strength and limitation of the study

This study aimed to explore behavioral components that serve as strong predictors of intention and behavior. Additionally, it introduced a new variable, the duration of marriage, which had not been included in previous studies conducted in Ethiopia. One limitation of the study was the presence of social desirability bias. To mitigate this bias, data collectors interviewed the women privately in a separate room.

## Data Availability

The original contributions presented in the study are included in the article/Supplementary Material, further inquiries can be directed to the corresponding author.
